# ‘It’s not like real therapy’: young people receiving child welfare services’ experiences of video consultations in mental healthcare in Norway: a mixed methods approach

**DOI:** 10.1186/s12913-023-09939-x

**Published:** 2023-09-05

**Authors:** Vibeke Krane, Jannike Kaasbøll, Silje L. Kaspersen, Marian Ådnanes

**Affiliations:** 1 Faculty of Health and Social Sciences, Department of Health, Social and Welfare Studies, University of South-Eastern Norway, Drammen, 3007 Norway; 2 Department of Mental Health, Faculty of Medicine and Health Sciences, Regional Centre for Child and Youth Mental Health and Child Welfare (RKBU Central Norway), Norwegian University of Science and Technology, Trondheim, Norway; 3 Department of Health Research, SINTEF Digital, Trondheim, Norway

**Keywords:** Child welfare service, Child and adolescent mental health services, Video consultation, Therapeutic relationship, User involvement

## Abstract

**Background:**

Video consultations has been suggested to lower the threshold for child and adolescent mental healthcare treatment. This study explores how young people receiving child welfare services experience video consultations in child and adolescent mental healthcare. The study is part of a larger Norwegian study of access to health services for this target group.

**Methods:**

The study has a mixed methods design including qualitative interviews and a quantitative survey, with young people receiving child welfare services. The qualitative interviews included 10 participants aged 15–19. The survey included 232 participants aged 16–24 of which 36 reported having received video consultations in mental healthcare. The interviews were analysed using thematic analysis. The survey data was presented as frequencies to clarify the distribution of positive and negative perceptions of video consultation.

**Results:**

The results show that the participants experienced video consultations as more superficial and less binding, compared to in-person sessions. They raised concerns of the therapeutic relationship, however some found it easier to regulate closeness and distance. In the survey several reported that their relationship with the therapist got worse, and that it was much more difficult to talk on screen. Moreover, a large proportion (42%) claimed that video consultations did not fit their treatment needs overall. However, a minority of the participants found it easier to talk to the therapist on screen.

**Conclusions:**

The study reveals important weaknesses and disadvantages of online therapy as experienced by young people receiving child welfare services. It is particularly worrying that their criticism involves the relational aspects of treatment, as children receiving child welfare services often have relational experiences which make them particularly sensitive to challenges in relationships. This study shows that youth involvement in decision making of video consultations in therapy has been rare. Clinicians should be aware of these young people’s doubts regarding the quality of video consultations in child and adolescent mental health care. Further studies should examine how user involvement can be incorporated in video consultations in therapy and how this could improve experiences and the quality of video consultations.

## Introduction

This study explores how young people receiving help from child welfare services (CWS) experience video consultations (VC) in mental health treatment. VC is a supplement or an alternative to in-person consultations, using live two-way video and/or audio interactions between therapist and client(s) or video conferences with interdisciplinary teams [1]. As a tool in child and adolescent mental health services (CAMHS), VC is far from new [2–5] and forms part of a broader array of eHealth services and digitalisation strategies used by health authorities in many countries. It is an important policy in Norway and a range of other countries to increase digitalisation of welfare and health services [6, 7]. However, VC was generally little used in CAMHS until COVID-19 necessitated a sudden change to digital communication [8, 9].

Young people receiving CWS have a higher prevalence of mental health problems due to difficult living conditions, trauma exposure, and underprivileged backgrounds [10–12]. Several studies show, paradoxically, that these young people often have poorer access to mental health services [12–17]. There has been a focus on how mental health services can reach this group, and VC has been suggested as an innovative solution [18, 19]. The concern that young people receiving CWS were particularly vulnerable during COVID-19 further accentuated the need for CAMHS to maintain contact with them [20–22].

Studies indicate that VC can improve access to specialist mental healthcare by lowering important barriers like geographical distance, low socio-economic background and poverty [18, 19, 23]. However, structural inequality in access to technological solutions and disparities in knowledge of digital tools have been highlighted [9, 21, 24, 25]. Digital solutions in CAMHS and CWS also pose confidentiality and safety risks as clients need a stable internet connection and privacy for a VC [20, 22, 26].

Several studies of service providers’ perspectives indicate promising results and positive experiences of VC in mental health treatment for young people [2, 18, 19, 23, 27]. The review by Boydell et al. [2] suggests that young people may be more likely to respond to VC and internet-based applications. A pilot study of PTSD treatment showed promising results in the use of VC [19]. Malas [23] found that primary care providers felt that a telepsychiatric collaborative programme, including VC, improved treatment to youth because of better access to specialist psychiatric assessments. Simpson et al. [27] argue that VC is an opportunity to develop therapeutic relationships with young people as they have grown up with technology. Archard and colleagues [28] did a service evaluation of the shift from face-to-face to remote forms of care from a single specialist CAMHS team during Covid − 19 with 16 vulnerable young people in residential care, foster care, adopted, or involved in justice services. They found a high level of satisfaction with the service and that the therapeutic relationship had a renewed significance when care was delivered remotely or as a hybrid solution. Nevertheless, half of the participants expressed a preference of face- to face contact when there was no need for physical distancing. Moreover, some clinicians have also warned about an existing digital divide due to socioeconomic inequalities and poverty, such as inequalities in access to internet and electronic devices for marginalized children [29]. Several concerns have also been raised regarding the development and quality of the therapeutic relationship in VC [5, 6, 21]. Establishing a therapeutic relationship in VC risks losing some of the nuances of communication related to body language, facial expressions and subtle social cues [9, 26, 30]. There are also concerns that therapists in VC have limited access to risk assessment of the child’s safety, care situation and need for CWS support. Therapists have also emphasised the difficulty of accessing children’s/adolescents’ developmental status and safeguarding welfare concerns using VC [20, 31].

During COVID-19, interdisciplinary collaboration in CAMHS and CWS was frequently modified to e-health solutions [1, 26, 31, 32]. Studies show that these solutions increase participation of professionals, encourage more frequent meetings and enhance continuity in collaboration. However, there is a need to explore how to include families with limited digital access in these meetings and to consider topics suitable for video conferences [1, 32].

VC in mental healthcare has been suggested to lower the threshold for CAMHS treatment for young people receiving CWS. Since the use of VC increased considerably during COVID-19, it is important to enhance knowledge of its potential consequences. Research on VC in CAMHS is still limited and most studies are based on therapists’ and professionals’ perspectives. Studies exploring the quality of VC are needed and young people’s own perspectives on VC are greatly under-researched. Against this background, this study will explore the views of young people receiving help from CWS perspectives’ on VC in CAMHS treatment. We pose the following research questions:

How do young people receiving CWS experience VC in mental health treatment?

How do young people receiving CWS experience the therapeutic relationship in VC treatment?

## Methods

This study is part of a larger project investigating access to health services for young people receiving CWS. The present study was directed at young people receiving CWS as well as CAMHS. The study has a mixed methods design including qualitative interviews and a quantitative survey [33].

### Study setting

The Norwegian CWS has a twofold mandate under the Child Welfare Services Act [34]: (1) to protect children from neglect and abuse, and (2) to provide help and support to ensure a secure and caring environment for children [35]. The day- to-day service is organized within each municipality and perform assistance including: home-based support, out-of-home placements, monitoring out-of-home placements, network meetings and aftercare for young people up to the age of 25 [36].

The Norwegian CAMHS consists of multidisciplinary treatment units that are part of the public, secondary specialized health care system. It is organised as a specialist service in regional hospitals and community based outpatient clinics for children and adolescents 0–18 years old (possible to extend the treatment until the age of 23). The aim of CAMHS is to assess and treat serious mental health problems and disorders. Most of patients are treated in community-based outpatient clinics where psychiatrists, psychologists, pedagogues, social workers and nurses work in interdisciplinary teams. CAMSH require a referral by doctor, a psychologist or child welfare worker [37].

### Mixed methods

Combining qualitative and quantitative methods in a mixed-method design can be helpful in understanding how complexity impacts on interventions in specific contexts [33]. A parallel-results convergent synthesis design [33] was used in collecting, analysing, and interpreting qualitative and quantitative data. We started the data collection conducting most of the qualitative interviews, followed by a preliminary analysis of these interviews which we used as inspiration to develop questions about VC in the survey. Qualitative and quantitative data are analysed and presented separately in the results section, and the results are interpreted in the discussion section.

### Qualitative interviews

**Participants and data collection.** The participants for the qualitative study were recruited via CWS in the municipalities and via information in social media (posted at Facebook of the Norwegian foster home association and Competence Center for Lived Experience and Service Development, and Instagram pages of organizations for children who receive CWS), and to some extent via ‘snowball sampling’ (based on referrals from participants).

The main project was conducted in 2021–2022 and included 27 qualitative interviews with young people who had received CWS and CAMHS. Ten of these participants had experiences with VC. The in-depth interviews with these ten young persons (aged 15–19) who had experience of VC in CAMHS are included in the present study. The participants are presented in Table 1, including information about gender, age, what kind of CWS assistance and their self-reported problems.

**Table 1:** Participants in the qualitative study

Pseudonym	Sex	Age (years)	Housing	CWS assistance	Self-reported mental health problems
Ariana	Female	18	Living alone in a studio flat	Aftercare	Depression, anxiety, insomnia, previous drug problems
Beth	Female	16	Living with parents	Receiving home-based support	ADHD, PTSD, depression, self-harm, suicidal thoughts, suicide attempts,
Cathy	Female	15	Foster care	Foster care	Eating disorder
Dan	Male	16	Institutional care	Institutional care	Borderline personality disorder, ADHD, suicide attempt
Eve	Female	17	Living alone in a studio flat	Receiving home-based support	Depression
Faith	Female	17	Foster care	Foster care	Anxiety, PTSD
Gail	Female	19	Living with friends in a flat	Aftercare	Depression, ADHD, psychosis, personality disorder, suicide attempt, drug problems
Heather	Female	18	Living with friends in a flat	Aftercare	Eating disorders, ADHD, borderline personality disorder, bipolar disorder, depression, chronically suicidal, anxiety, dissociative disorder
India	Female	17	Living with parents	Receiving home-based support	Eating disorders, self-harm, suicide attempts
Jude	Female	19	Living in sheltered housing	Institutional care	PTSD, anxiety, depression

The participants could choose if they wanted to do the interview in-person or online. Participants were asked about their experiences of VC in questions like: ‘Have your received VC from CAMHS?’ and ‘What did you think about having VC with CAMHS?’ and ‘What is the difference between VC and in-person consultations?’

**Qualitative analysis.** The qualitative interviews were transcribed verbatim. All transcripts were read by two of the researchers and the ten interviews that included experiences of VC were subject to thematic analysis inspired by Braun and Clarke [38] and Clarke et al. [39]. This analysis was based on a constructivist epistemological assumption. Thus we acknowledge that knowledge is interpreted and developed in dialogues and interactions between people [40]. All text was coded in NVivo software, and sub-themes were developed from these codes and discussed and revised by two of the researchers. Agreement on the analytical themes was reached via internal meetings in the research group discussing data and analyses. This led to three main themes, as presented in the results.

### Quantitative survey

**Sample.** An invitation to participate in an online survey was sent via email to managers of all municipal child welfare services in Norway (n = 221) and to managers of all child care institutions registered at Bufetat (n = 136). The managers were asked to forward the invitation, including a link to an online questionnaire, to the target group via their usual means of communication with them (email, SMS, or other ways). The questionnaire was accessible online between May to November 2022. Two reminders about distributing the survey to the target group was sent to the CWS during the data collection period.

**Data-collection**. Demographic information included age, gender (female, male or ‘other’ ) and living situation (i.e. ‘I live with both my parents in one place; ‘I change which of my parents I live with’; ‘I only live with one of my parents’; ‘I live with family members other than my parents’; ‘I live in a residential youth care institution’; ‘I live in emergency shelters’; ‘I live with foster parents’; ‘I live with adoptive parents’; ‘I live with friends/collectives’; ‘I live alone/in a dormitory’; (yes/no response categories) and ‘Other, describe’ (open end question).

Based on a preliminary analysis of the qualitative interview data and previous literature [41], we focused the survey on experiences of the therapeutic relationship. The questions about VC use have previously been used by Gullslett et al. [41]. The survey included 15 specific questions about receiving VC from CAMHS. Respondents were asked to think about their experiences from meeting their therapist via VC when answering the questions. One initial question was used to identify participants with experience of VC: ‘In the past two years, have you received video consultations in connection with treatment services in CAMHS or mental healthcare for adults?’ (Yes/No). Other background variables concerning the use of VC included the following questions: ‘Did you start treatment on screen because it was closed for regular attendance due to the COVID-19 pandemic?’ (Yes/No/I don’t know); ‘How many times have you had on-screen treatment?’ (Once/2–5 times/6–9 times/10 times or more); ‘Have you had an appointment with physical attendance in addition to digital treatment?’ (Yes/No); ‘Where were you during video consultations?’ (At home/At school/Other).

Questions about experiences with VC included: ‘Do you think it’s okay to see yourself on screen? (Yes/No/I don’t know); ’Is the relationship with your therapist the same, or better or worse when you are on a screen than in regular attendance?’ (Much better/A little better/The same/Slightly inferior/Much worse/Not Applicable); ‘Can you say what you want to say on the screen?’ (To a very large extent/To a large extent/To some extent/To a small extent/Not at all); ‘Talking to the therapist on screen, do you find that it is the same, easier, or more difficult than in person?’ (Much easier; A little easier/The same/A little harder/Much harder/Not Applicable). In addition, five items were included: ‘Do you feel like the therapist is listening to you on screen?’ 2)’ Do you find that the therapist understands what you are conveying on screen?’ 3) ‘Do you find it difficult to concentrate on the topics you talk about on screen?’ 4) ‘Does digital/on-screen therapy fit your therapy needs?’ 5) ‘All in all, are you satisfied with the digital/on-screen therapy?’. The responses to these questions were given on a 5-point Likert scale, where 1 = To a very large extent, 2 = To a large extent, 3 = To some extent, 4 = To a small extent, and 5 = Not at all.

**Quantitative analysis**. Quantitative data were analysed using IBM® SPSS® Statistics. Descriptive analysis included measures of frequencies.

### Ethics approval and consent to participate

The data collection was ethically approved by ‘Regional Committees for Medical and Health Research Ethics’ (reference IDs: 426536) and ‘Sikt - Norwegian Agency for Shared Services in Education and Research’ (reference IDs: 871209). All participants received written and oral descriptions of the study. Oral informed consent was obtained from all participants. Parental/guardian consent was obtained for participants under 16 years old. All research methods followed the relevant guidelines and regulations.

### User involvement

A young person with user experience from CWS was involved in the planning of the project, recruitment of participants and in developing the interview guide. Furthermore, the project collaborated closely with a professional advisor at a competence center for lived experience and service development in recruitment of participants and conduction seven of the interviews.

## Results

The qualitative analysis resulted in three main themes: (1) Video consultations can be OK, but it’s not like real treatment, (2) You can escape video consultations when it’s too demanding, and (3) Video consultations can be timesaving but is can also be really messy.

### Video consultations can be OK, but it’s not like real treatment

The participants reported having used different digital platforms from their computer or mobile phone in VC with CAMSH both during and after COVID − 19 lockdown. Several participants described that VC did not meet their needs for therapy and support, and that they generally found the quality of VC poorer than in-person sessions. VC was frequently described by the participants as an alternative to what the informants described as *real* (in-person) CAMHS sessions with their therapist. Several expressed that they preferred in-person sessions as the ‘real thing’. Eve (17 years) talked about her experiences with her psychologist during the lockdown:

During the lockdown, I had therapy sessions with a psychologist. We frequently used the phone, but after a while I went there and had real sessions.

She described finding in-person sessions more demanding but considered them more useful for that very reason. The informants explained that they found the quality of VC poorer than in-person sessions as it limited the therapeutic interactions. Gail (19 years), expressed her scepticism of VC:

Well, I’d say it’s not as good as usual sessions… when we meet face-to-face. Because you talk to a screen… it’s not just the digital aspect that decides whether the session is positive or not… But I think for most people it’s best to meet in person. For some it could be OK to have VC.

Some of the participants said that VC could work if they knew the therapist from before, however they still preferred in-person sessions. Several found the VC more superficial and highlighted that the therapists were not able to read their mood in the same way as in in-person sessions. Ariana (18 years) explained how consultations with her psychologist were moved to Skype during the first COVID-19 lockdown. She knew her psychologist well and liked her, and therefore felt that the VC worked OK. However, she also felt that VC limited useful therapeutic interactions between her and her therapist, much because she thought VC miss out on important body language. She explained:

When I meet her in person, I think it’s easier for her to … well, you see each other on video, but I think when we’re in the same room it’s easier to read each other’s body language. Then she’ll notice when I’m feeling sad, if I’m angry or things like that… and if for example I suddenly start to lie about how I’m doing.

### You can escape video consultations when it’s too demanding

While in the theme presented above, in-person sessions were described as more demanding and therefore preferable, other participants highlighted VC as a way out of difficult and demanding therapy sessions. Some felt that VC allowed them to regulate closeness and distance in the sessions. Several participants described how they had logged off from digital sessions when they found the sessions too demanding. Heather (18 years) described how VC offered an opportunity to withdraw from the session: *‘It was kind of OK, because I just hung up when I didn’t want to talk any more….Like…enough of this. I can’t take anymore’.* Both she and Dan (16 years), found it easier to end a VC than face-to-face sessions sitting with the therapist at CAMHS.

Several participants had experience of interdisciplinary collaborative meetings on digital platforms. Those involved were typically health and social care staff, the CAMHS therapist, CWS caseworkers, foster parents, parents, teachers, and educational support staff. These meetings were often moved online during COVID-19, and some participants found that these meetings were now permanently held on digital platforms. Several reported finding interdisciplinary meetings demanding. It was difficult to talk about personal problems with many people in such a large forum, however the digital solution had given some an opportunity to escape when it was too demanding. Faith (17 years) had found a way to hide from the camera in these large meetings. She explained:

Luckily we don’t have the meetings in a room … we join in Teams or Zoom… … It’s a bit like … I can hide from the camera… It’s been like that because of Covid. But now we might actually have to meet in person, they haven’t said that I have to join, but it is expected of me….

Another girl, India (17 years), explained how she withdrew from digital collaborative meetings when she did not want to participate. She just turned off the computer and thus left meetings when she found them too demanding.

### Video consultations can be timesaving but is can also be really messy

Some participants talked about how VC could be a practical solution for long distances and saving travel time, however they also had some important reservations. Dan (16 years) who had lived in several childcare facilities for a long time expressed concern about the use of VC for young people living in institutional care. He was worried that if CAMSH sessions were frequently transferred to VC, young people living in childcare facilities could be more isolated in the institutions. Furthermore, he was concerned that the employees in such facilities would prefer VC as a solution due to practical reasons and limited resources. He explained:

Because where I used to live, it took an hour to get to CAMHS… And after COVID, they found out that it works just as well doing video consultations. And those sessions wouldn’t have any effect on me.

He also highlighted how VC must be voluntary and that young people should be entitled to choose the method themselves. He explained:

It could be quite frightening to start using video consultations, unless the person decides for themselves… Because it’s ok if they choose it themselves. But if people think: ‘This is just a practical solution, because this person shouldn’t be allowed to go out’ then it’s a rather scary way to go.

None of the participants mentioned any challenges with access to digital tools or handling digital platforms. However, poor internet connection during VC-sessions and meetings was highlighted as a problem by several. Jude (19 years) described how a bad internet line could complicate meetings and thus preferred in-person meetings:

*Well, when we have VC - and more than two people participate, the internet line is really bad. Someone starts talking, someone else can’t hear - and then they talk at the same time - ….and then they have to repeat themselves, and the child welfare person starts to talk because they didn’t hear what the other one said. So then it gets very messy. But it’s a good thing after all instead of driving for nine hours. So it’s an easy way to solve it if it’s at short notice… But it’s also really messy.*

In summary, the findings show that the young participants receiving CWS and CAMHS expressed scepticism and raised several concerns about the use of VC. Their main objection to VC seemed to be the quality of their interaction with the therapist. While several described VC as less binding and demanding than in-person sessions, some appreciated how VC gave them a new possibility to regulate closeness and distance. All participants preferred in-person consultations to VC.

## Results from the survey

### Background characteristics

A total of 232 people aged 16–23 responded to the questionnaire sent via CWS and child care facilities throughout the country. Of these, 36 participants (15%) reported having received digital health services/VC in treatment in CAMHS or mental healthcare for adults. Of these 36, 69% (n = 25) were girls, 28% (n = 10) were boys, and 3% (n = 1) identified as ‘other’. The mean age of this group was 19 years (SD = 1.78, range 16–24). Six of them lived in a childcare facility, ten lived with foster parents, four with one or both parents, four with friends, in a group or with another family member, eight lived alone in studio flats, and three in other kinds of housing. One respondent did not reply to this question. As shown in Tables 2 and 27 (75%) participants stated that they started online therapy because COVID-19 prevented face-to-face sessions. Most participants (41%) had had online sessions 2–5 times, and 81% reported having had these sessions at home.

**Table 2:** Frequency distribution of responses to questions about starting VC, the number of VC sessions, and the context of VC by participants aged 16–24 receiving CWS (n = 36)

		n	Percent	Valid Percent
Did you start video consultations because face-to-face consultations were impossible during COVID-19?				
	Yes	27	75	75
	No	8	22	22
	I don’t know	1	1	3
	Missing values	3	2	
How many times have you had video consultations?				
	Once	8	22	24
	2–5 times	14	39	41
	6–9 times	2	6	6
	10 times or more	10	28	29
	Missing values	2	5	
Where were you during video consultations?				
	At home	29	80	88
	At school	2	6	6
	Somewhere else	2	6	6
	Missing values	3	8	

### Experiences with digital treatment/video consultations

As presented in Table 3, when asked if they thought it was okay to see themselves on screen,

**Table 3:** Frequency distribution of the experiences of VC of participants aged 16–24 receiving CWS (n = 36)

		n	Percent	Valid Percent
Do you think it’s okay to see yourself on screen?				
	Yes	13	36	40
	No	11	31	33
	I don’t know	9	25	27
	Missing values	3	8	
In video consultations, is your relationship with your therapist the same, better or worse than in face-to-face consultations?				
	Much better	0	0	0
	A little better	0	0	0
	The same	12	33	36
	Slightly worse	10	28	30
	Much worse	4	11	12
	Not applicable	7	20	22
	Missing values	3	8	
Can you say what you want to say in video consultations?				
	To a very large extent	4	11	12
	To a large extent	7	20	21
	To some extent	8	22	24
	To a small extent	12	33	37
	Not at all	2	6	6
	Missing values	3	8	
When you talk to your therapist in video consultations, do you find it easier, the same, or harder than in person?				
	Much easier	2	5	6
	A little easier	4	11	12
	The same	8	22	24
	A little harder	6	17	18
	Much harder	10	29	31
	Not applicable	3	8	9
	Missing values	3	8	

The responses were relatively evenly divided between yes, no and don’t know. No participants said that their relationship with the therapist was ‘a little better’/much better’ than in regular sessions. About one third of participants rated their relationship with their therapist as ‘the same’, while 39% rated it as ‘worse’. In addition, about 40% of respondents had some difficulty expressing themselves in VC, while about 30% did not. Participants reported finding it harder to talk to the therapist during VC than in person, compared with those who found it easier (45% versus 34%).

**Fig. 1:**
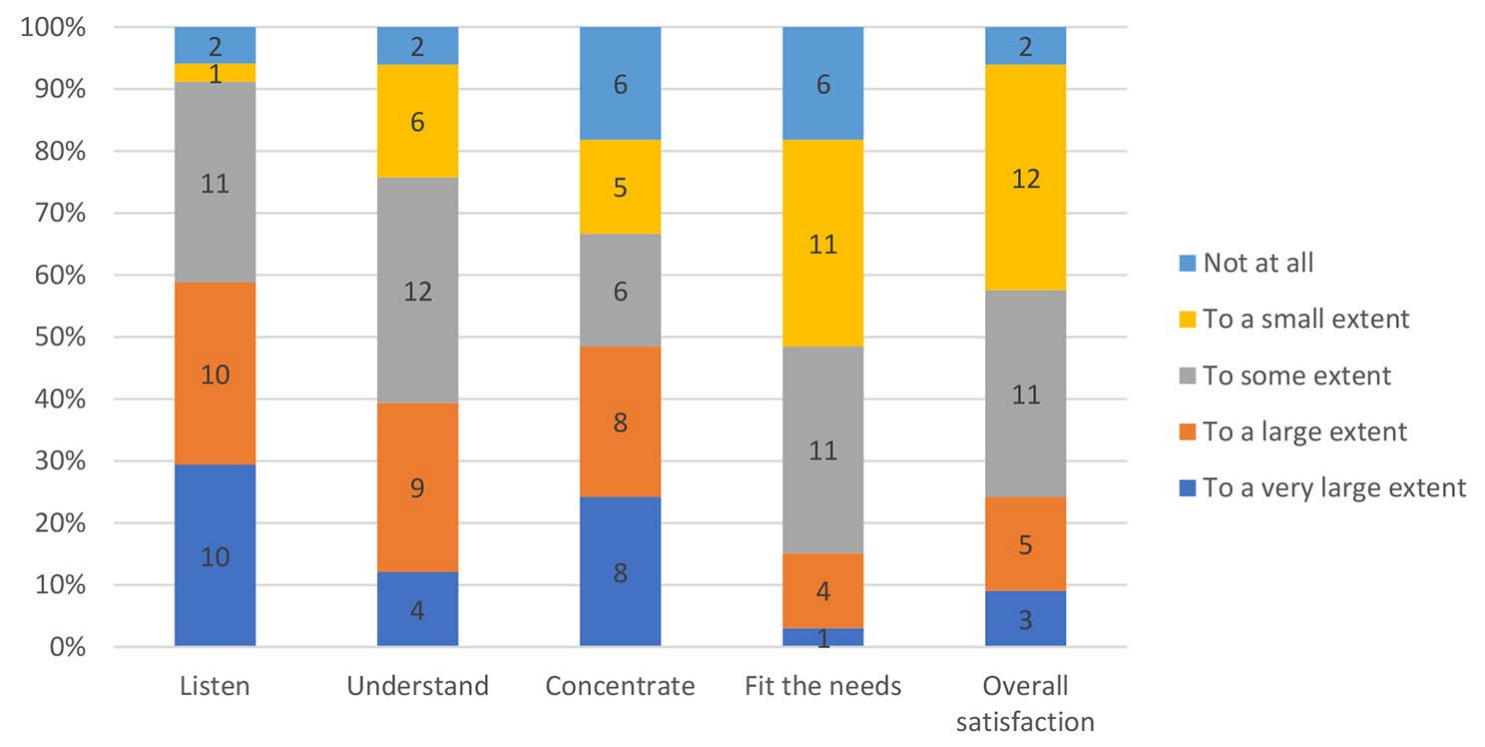
Distribution of responses on five items measuring satisfaction with treatment via video consultations

Figure 1 shows that a larger proportion of the participants reported clearly negative (36%) than clearly positive (24%) overall satisfaction with VC/online therapy. Most of the participants agreed with the statement that they felt their therapist was listening to them in VC. About 10% disagreed. As for concentration, about 20% rated their concentration in VC as very poor, and the same proportion found it unsuitable for their needs.

## Discussion

The qualitative interviews show that young people receiving CWS found that VC was more superficial and committed them less that in-person sessions with their therapist. The survey confirms this, showing that many reported a poorer relationship with their therapist and greater difficulty in talking in VC. Our discussion deals with the therapeutic relationship in VC, components of the therapeutic relationship and user involvement in decision making in VC.

### The therapeutic relationship in Video Consultations

There is consistent evidence that the quality of the therapeutic relationship is crucial for the success of psychotherapy [42]. Moreover, evidence shows that positive, supportive relationships with adults, both therapeutic and non-therapeutic, can enhance outcomes for adolescents receiving CWS [43] Studies of psychotherapy even describe the therapeutic relationship as having a stronger association with positive outcomes for those adolescents with the poorest attachment history [44]. However, these young people may show considerable ambivalence towards help for emotional problems [28]. Hence, establishing and maintaining positive therapeutic relationships plays a vital role in the experiences of VC for young people receiving CWS. Our survey shows that 45% of the respondents found it harder to talk to the therapist online than in in-person meetings. Moreover, 39% felt that their relationship with the therapist was worse, and none found it better in VC. The qualitative findings are in line with these results, showing that the participants had serious concerns about the quality of VC and described how their relationship with the therapist committed them less. These findings contrast somewhat with those from several other studies that have indicated good quality and positive effects of VC [2, 18, 19, 23, 27]. However, most of those studies included only service providers’ perceptions, not clients’ own experiences, and potential clinical implications has only been measured in a small pilot study by Stewart et al. A limitation of pilot studies is potential self-selection bias, as they often attract digitally optimistic and mature participants [41]. By contrast, most participants in our survey and all participants in our qualitative study had experienced VC during COVID-19 restrictions. This may have led to more ad-hoc solutions with technical problems and unprepared therapists being thrown into VC therapy. Moreover, some of the participants may have been living in greater social isolation than in normal circumstances and were perhaps particularly vulnerable during COVID-19. However, our rather homogenous findings of these young peoples’ experiences of VC raise questions about the quality of the therapeutic relationship in VC [5, 6, 21].

### Experiences of therapeutic components in Video Consultations

Essential components for establishing positive therapeutic relationships are empathy, genuineness and engagement [45]. Our participants’ description of VC as ‘not real therapy’ resembles findings of therapists’ descriptions of VC as ‘a filter for emotions’ [6]. Other studies have also pointed out that subtle cues, facial expressions and body language that might be essential for showing empathy, genuineness and engagement could be lost in virtual communication [9, 26]. Moeller’s [46] review of adults’ experiences of VC is in line with this finding: all of the included studies found that patients mentioned that VC contact with the therapist was less personal and intense than in person. Studies have also revealed that participants found it easier to switch to VC when they had already established a therapeutic relationship with the therapist and met them in person [9, 18, 46]. Several studies show that a combination of in-person meetings and VC may be useful [18, 20, 28]. Most of the participants in our survey had received both VC and in-person sessions and all participants in the qualitative study had a relationship with the therapist prior to VC. Some of our qualitative findings indicate that knowing the therapist made it a little easier to switch to VC. Overall, however, our data show that our participants preferred face-to-face sessions. Many found it more difficult to connect with the therapist in VC, while some emphasised that they preferred in-person therapy because they found it more demanding. This shows that some of these young people were aware of how demanding therapy could be in order to be useful for them, and therefore appreciated the in-person interactions and genuine meetings with the therapist.

A minority of the participants reported finding it easier or much easier to talk to the therapist in VC. This could be in line with the qualitative findings where our participants explained how VC could be a way of hiding, implying that the client could regulate closeness and distance in the therapeutic context. As discussed above, therapy can be demanding, and for some of the participants in-person therapy might be too difficult. Maybe VC as ‘a filter for emotions’ [6] is just what they need. Other studies have found that young patients with autism found contact with the therapist in VC less personal and intense than in person [46]. Stabler et al. [30] discussed how the possibility of ‘hanging up’ can be understood as a powerful tool and a way of taking control of the terms of their engagement in sessions. When some of our participants also described how they ‘hung up’ in VC sessions when they had had enough, this could be understood as a way of taking control and drawing a line for closeness in therapy. The issue of the subjective sense of closeness and distance in therapy and the association with people’s different attachment styles has been addressed [47]. It is well documented that young people in CWS often have experienced several losses in important relationships prior to and upon entry to the system [43]. These experiences often lead to relational challenges that can influence their ability to become involved in close relationships. Moreover, young people receiving CWS have poorer access to mental health services [12, 13], and thus any approach that could be useful in reaching some of this group should be welcomed. The study of the experiences of young people with autism of remote psychological interventions pointed out that VC reduced the intensity of the social interaction and made it more controllable [46]. Although a high proportion of our participants had negative experiences of therapeutic relationships in VC, it could be argued that VC has the potential to be a way of regulating closeness and distance, which could be positive for some of these young people.

### User involvement in Video Consultation decision-making

Our findings show little evidence of user involvement in decisions about using VC as a therapeutic approach. In the qualitative data the participants described VC as an ad-hoc solution to COVID-19 restrictions, and shared decision making was thus not an issue. Moreover, our findings reveal that a high proportion of the participants reported that VC did not meet their therapeutic needs. Some of the qualitative findings even showed that VC was felt to be particularly inadequate during the restrictions of the COVID-19 crisis. User involvement is crucial in working with young people receiving CWS as they are often particularly vulnerable in terms of engagement in relationships [43]. It is emphasised as important for helpers to transfer power to these young people, because trusting relationships are necessary for them to feel safe in asking for help [48]. Client engagement is crucial in therapy [45] and the feeling that VC did not meet our participants’ needs represents a serious threat to the effect of VC therapy. Despite this, VC might have a potential for some young people receiving CWS. One way of involving users in therapy is a feedback-informed approach to create continuous feedback loops between clients and therapists to enable clients to be explicitly involved in therapeutic decision making. This approach has shown beneficial outcomes for youth [49]. Collecting feedback and using shared decision making increase the likelihood that VC will have a positive therapeutic effect for some young people. As these young people have different needs, and their preferences may vary over time [50], the preferences of using VC should be explored in therapy. However, there are particular pitfalls in providing VC therapy to young people in CWS: it is important to be aware of the risk of being unable to uncover serious neglect and abuse when therapy is provided online [20, 35]. This risk must be considered if VC is chosen as the preferred therapeutic approach and it will be important that other service providers meet these young people in person to ensure that serious risks in their lives are uncovered.

### Strengths and limitations

The present study is one of the first to report the experiences with VC in CAMHS of young people receiving CWS. The mixed method design gave the researchers the flexibility to use both quantitative and qualitative data to elicit useful findings about the population.

Even though the ten participants in the qualitative study struggled with various problems, came from different parts of Norway and received different CWS and CAMHS, they shared many similar experiences of VC. This suggests that the interviews provided a complementary and coherent picture of these clients’ experiences and the meanings they attached to them. All participants in the qualitative study had experience of VC during COVID-19 restrictions, and the possible impact of this factor is discussed above.

The small sample size, particularly regarding male respondents (28% in the survey and n = 1 in the interviews), limited the generalisability of the results and possibilities for multivariate analysis of the quantitative data. It is well known that there are gender differences in the use of mental healthcare services, where males are underrepresented [51]. In future studies, it will be crucial to examine whether there are distinct gender differences in the effects of VC in CAMHS for children and adolescents who receive CWS. It is also a limitation that there are no validated measurement instruments available to assess patient satisfaction with VC in adolescents.

## Conclusion

While VC has been proposed as a possibility to lower the threshold for CAMHS treatment of young people receiving CWS, our study reveals important weaknesses and disadvantages of online therapy as experienced by clients in the target group themselves. It is particularly worrying that their criticism involves the relational aspects of treatment, as children receiving CWS often have relational experiences which make them particularly sensitive to challenges in relationships. Moreover, a positive therapeutic relationship is the key element in psychological treatment. More widespread use of VC treatment without further exploring this key element which lays the foundation for therapy could even create new barriers, despite the aim to reduce barriers to receiving treatment and to ensure continuity of treatment. It is important that VC does not become a part of standard therapy interventions that do not meet young person’s needs nor involve them in decision-making.

Most experiences of VC have been due to the COVID-19 restrictions and may explain why youth involvement in decision making has been rare. As it has been suggested to increase the use of VC in CAMHS it is important to further explore the experiences of VC of young people in CWS in order to inform suggested strategies. Further studies should examine how user involvement can be incorporated into VC therapy and how this could improve experiences and quality of VC.

## Data Availability

Qualitative data extracts are presented in the article to support the findings. The data generated and analysed during the current study are not publicly available as the data collected is sensitive and could compromise the confidentiality and anonymity of the participants but are available (limited) from the corresponding author on reasonable request. The quantitative datasets used and/or analysed during the current study available from the corresponding author on reasonable request.

## References

[1] Coon JC, Bush H, Rapp JT. Eight months of Telehealth for a state-funded project in Foster Care and Related Services: Progress made and Lessons learned. Behavior Analysis in Practice; 2022. 10.1007/s40617-022-00682-z PMC8924942 35313702

[2] Boydell KM, Hodgins M, Pignatiello A, Teshima J, Edwards H, Willis D (2014). Using technology to deliver mental health services to children and youth: a scoping review. J Can Acad Child Adolesc Psychiatry.

[3] Chakrabarti S (2015). Usefulness of telepsychiatry: a critical evaluation of videoconferencing-based approaches. World J Psychiatry.

[4] Pesämaa L, Ebeling H, Kuusimäki M-L, Winblad I, Isohanni M, Moilanen I (2004). Videoconferencing in child and adolescent telepsychiatry: a systematic review of the literature. J Telemed Telecare.

[5] Slone NC, Reese RJ, McClellan MJ (2012). Telepsychology outcome research with children and adolescents: a review of the literature. Psychol Serv.

[6] Gullslett MK, Kristiansen E, Nilsen ER (2021). Therapists’ experience of Video Consultation in Specialized Mental Health Services during the COVID-19 pandemic: qualitative interview study. JMIR Hum Factors.

[7] Nordesjö K, Scaramuzzino G, Ulmestig R (2022). The social worker-client relationship in the digital era: a configurative literature review. Eur J Social Work.

[8] Patel R, Irving J, Brinn A, Broadbent M, Shetty H, Pritchard M (2021). Impact of the COVID-19 pandemic on remote mental healthcare and prescribing in psychiatry: an electronic health record study. BMJ Open.

[9] Worsley J, Hassan S, Nolan L, Corcoran R (2022). Space to hide’: experiences of remote provision across child and adolescent mental health services (CAMHS). BMC Health Serv Res.

[10] Lehmann S, Havik OE, Havik T, Heiervang ER (2013). Mental disorders in foster children: a study of prevalence, comorbidity and risk factors. Child Adolesc Psychiatry Mental Health.

[11] Lehmann S, Kayed NS (2018). Children placed in alternate care in Norway: a review of mental health needs and current official measures to meet them. Int J Social Welf.

[12] Jozefiak T, Kayed NS, Rimehaug T, Wormdal AK, Brubakk AM, Wichstrøm L (2016). Prevalence and comorbidity of mental disorders among adolescents living in residential youth care. Eur Child Adolesc Psychiatry.

[13] Larsen M, Baste V, Bjørknes R, Myrvold T, Lehmann S (2018). Services according to mental health needs for youth in foster care?–A multi-informant study. BMC Health Serv Res.

[14] Besier T, Fegert JM, Goldbeck L (2009). Evaluation of psychiatric liaison-services for adolescents in residential group homes. Eur Psychiatry.

[15] Bradford S, Rickwood D (2012). Psychosocial assessments for young people: a systematic review examining acceptability, disclosure and engagement, and predictive utility. Adolesc Health Med Ther.

[16] González-García C, Bravo A, Arruabarrena I, Martín E, Santos I, Del Valle JF (2017). Emotional and behavioral problems of children in residential care: screening detection and referrals to mental health services. Child Youth Serv Rev.

[17] Burns BJ, Phillips SD, Wagner HR, Barth RP, Kolko DJ, Campbell Y (2004). Mental health need and access to mental health services by youths involved with child welfare: a national survey. J Am Acad Child Adolesc Psychiatry.

[18] Mundt AP, Irarrázaval M, Martínez P, Fernández O, Martínez V, Rojas G. Telepsychiatry consultation for primary care treatment of children and adolescents receiving child protective services in Chile: mixed methods feasibility study. JMIR Public Health and Surveillance. 2021;7(7). 10.2196/25836 PMC8367295 34292164

[19] Stewart RW, Orengo-Aguayo RE, Cohen JA, Mannarino AP, de Arellano MA (2017). A pilot study of trauma-focused cognitive-behavioral therapy delivered via Telehealth Technology. Child Maltreat.

[20] Eapen V, Dadich A, Balachandran S, Dani A, Howari R, Sequeria AZ (2021). E-mental health in child psychiatry during COVID-19: an initial attitudinal study. Australasian Psychiatry.

[21] Loria H, McLeigh J, Wolfe K, Conner E, Smith V, Greeley CS et al. Caring for children in foster and kinship care during a pandemic: lessons learned and recommendations. J Public Child Welf. 2021.

[22] Cummings A (2023). The views of Mental Health Professionals who Use Digital Methods to support care-experienced Young People. Practice.

[23] Malas N, Klein E, Tengelitsch E, Kramer A, Marcus S, Quigley J (2019). Exploring the Telepsychiatry experience: primary care provider perception of the Michigan Child Collaborative Care (MC3) program. Psychosomatics.

[24] Chen JA, Chung W-J, Young SK, Tuttle MC, Collins MB, Darghouth SL (2020). COVID-19 and telepsychiatry: early outpatient experiences and implications for the future. Gen Hosp Psychiatry.

[25] Conrad JB, Magsamen-Conrad K (2022). Understanding the impact of the coronavirus pandemic on families involved in the child welfare system: Technological capital and pandemic practice. Child & Family Social Work.

[26] Krane V, Ausland LH, Andvig E (2021). «Kan vi hjelpe når krisa rammer?»Barnevern, smittevern og store forskjeller i tjenestene under covid-19-pandemien. Tidsskrift for Velferdsforskning.

[27] Simpson S, Richardson L, Pietrabissa G, Castelnuovo G, Reid C (2021). Videotherapy and therapeutic alliance in the age of COVID-19. Clin Psychol Psychother.

[28] Archard PJ, Kulik L, Fitzpatrick SM, Awhangansi S, Moore I, Giles E et al. Young people’s views on specialist mental healthcare and remote delivery during the COVID-19 pandemic. Mental Health Practice. 2022.

[29] Aisbitt GM, Nolte T, Fonagy P, Editorial, Perspective (2023). The digital divide – inequalities in remote therapy for children and adolescents. Child Adolesc Mental Health.

[30] Stabler L, Cunningham E, Mannay D, Boffey M, Cummings A, Davies B, et al. I probably wouldn’t want to talk about anything too personal’: A qualitative exploration of how issues of privacy, confidentiality and surveillance in the home impact on access and engagement with online services and spaces for care experiences young people. Adoption & Fostering; 2023. 10.1177/03085759231203019 PMC10590277 37873026

[31] Archard PJ, Fitzpatrick S, Morris N, O’Reilly M (2022). Consultation in a specialist Mental Health Team for Vulnerable Children before and during the early stages of the COVID-19 pandemic: audit findings and practice-based reflections. Practice.

[32] Baginsky M, Manthorpe J. Multiagency working between children’s social care and schools during COVID-19: case study experiences from English local authorities and international reflections. J Integr Care. 2021.

[33] Noyes J, Booth A, Moore G, Flemming K, Tunçalp Ö, Shakibazadeh E (2019). Synthesising quantitative and qualitative evidence to inform guidelines on complex interventions: clarifying the purposes, designs and outlining some methods. BMJ Global Health.

[34] Ministry of Children and Families. Division of responsibility in the Child Welfare Services2021. Available from: https://www.regjeringen.no/en/topics/families-and-children/child-welfare/allocation-of-responsibilities-related-to-c1/id2353984/.

[35] Krane V, Ausland LH, Andvig E, Klevan T (2021). Business as Usual in Unusual Times: an explorative study of norwegian child welfare workers’ experiences during the COVID-19 pandemic. J Comp Social Work.

[36] The Norwegian Directorate for Children YaFA. The Norwegian Child Welfare Services (barnevernet). Available from: https://www.bufdir.no/en/child-welfare-services/.

[37] Ruud T, Friis S (2021). Community-based Mental Health Services in Norway. Consortium Psychiatricum.

[38] Braun V, Clarke V (2022). Conceptual and design thinking for thematic analysis. Qualitative Psychol.

[39] Clarke V, Braun V, Hayfield N (2015). Thematic analysis. Qualitative Psychology: A Practical Guide to Research Methods.

[40] Crotty MJ. The foundations of social research: Meaning and perspective in the research process. The foundations of social research. 1998:1-256.

[41] Gullslett MK, Silsand L, Breivik E, Nilsen ER. Implementing and Learning to Use Video Meetings in Mental Health Hospital Departments. The Nineteenth International Conference on Networking and Services, ICNS 2023; Barcelona, Spain2022.

[42] Wampold BE, Imel ZE (2015). The great psychotherapy debate: the evidence for what makes psychotherapy work.

[43] Curry A (2019). If you can’t be with this client for some years, don’t do it: exploring the emotional and relational effects of turnover on youth in the child welfare system. Child Youth Serv Rev.

[44] Zack SE, Castonguay LG, Boswell JF, McAleavey AA, Adelman R, Kraus DR (2015). Attachment history as a moderator of the alliance outcome relationship in adolescents. Psychother (Chic).

[45] Horvath AO (2001). The alliance. Psychother Theory Res Pract Train.

[46] Moeller AM, Christensen LF, Hansen JP, Andersen PT (2022). Patients’ acceptance of video consultations in the mental health services: a systematic review and synthesis of qualitative research. Digit HEALTH.

[47] Wiseman H, Atzil-Slonim D (2018). Closeness and distance dynamics in the therapeutic relationship. Developing the therapeutic relationship: integrating case studies, research, and practice.

[48] Riise A, Paulsen V. Facilitating Participation for Youths in Child Welfare Services in Transition to Adulthood: Practice between Formalities and Empowerment. Child Care in Practice. 2022:1–14.

[49] Tam HE, Ronan K (2017). The application of a feedback-informed approach in psychological service with youth: systematic review and meta-analysis. Clin Psychol Rev.

[50] Archard PJ, Awhangansi S, Moore I, Majumder P, Lewis M, O’Reilly M et al. Remote and Digitally Delivered Mental Health Support for Care-Experienced Young People: Some Practice-Based Reflections in Response to Cummings (2023). Practice. 2023:1–10.

[51] Campbell OLK, Bann D, Patalay P (2021). The gender gap in adolescent mental health: a cross-national investigation of 566,829 adolescents across 73 countries. SSM Popul Health.

